# Does CT colonography have a role for population-based colorectal cancer screening?

**DOI:** 10.1007/s00330-012-2449-7

**Published:** 2012-05-02

**Authors:** Margriet C. de Haan, Steve Halligan, Jaap Stoker

**Affiliations:** 1 Department of Radiology, G1-228, Academic Medical Centre Amsterdam, PO Box 22700, 1100 DE Amsterdam, The Netherlands; 2 Centre for Medical Imaging, University College London, London, UK

**Keywords:** CT colonography, Screening, Colorectal cancer, Population-based, Role

## Abstract

Colorectal cancer (CRC) is the second most common cancer and second most common cause of cancer-related deaths in Europe. CRC screening has been proven to reduce disease-specific mortality and several European countries employ national screening programmes. These almost exclusively rely on stool tests, with endoscopy used as an adjunct in some countries. Computed tomographic colonography (CTC) is a potential screening test, with an estimated sensitivity of 88 % for advanced neoplasia ≥10 mm. Recent randomised studies have shown that CTC and colonoscopy have similar yields of advanced neoplasia per screened invitee, indicating that CTC is potentially viable as a primary screening test. However, the evidence is not fully elaborated. It is unclear whether CTC screening is cost-effective and the impact of extracolonic findings, both medical and economic, remains unknown. Furthermore, the effect of CTC screening on CRC-related mortality is unknown, as it is also unknown for colonoscopy. It is plausible that both techniques could lead to decreased mortality, as for sigmoidoscopy and gFOBT. Although radiation exposure is a drawback, this disadvantage may be over-emphasised. In conclusion, the detection characteristics and acceptability of CTC suggest it is a viable screening investigation. Implementation will depend on detection of extracolonic disease and health-economic impact.

*Key Points*

*• Meta-analysis of CT colonographic screening showed high sensitivity for advanced neoplasia ≥10mm.*

*• CTC, colonoscopy and sigmoidoscopy screening all have similar yields for advanced neoplasia.*

*• Good quality information regarding the cost-effectiveness of CTC screening is lacking.*

*• There is little good quality data regarding the impact of extracolonic findings.*

*• CTC triage is not clinically effective in first round gFOBT/FIT positives.*

## Introduction

In Europe, colorectal cancer (CRC) is the second most common cancer, as well as the second most common cause of death from cancer [1]. In 2008, approximately 432,414 European citizens received a new diagnosis of CRC [2]. The majority of cancers develop from adenomatous polyps, benign precursors with a relatively long premalignant phase [3]. Adenomas can vary in size but those ≥10 mm and/or with ≥25 % of villous histology and/or high-grade dysplasia have the strongest association with malignancy and are named advanced adenomas [3]. It is estimated that the adenoma to carcinoma transition takes at least 10 years [4]. Several studies have shown that removal of adenomas (e.g. via a CRC screening programme) results in reduced CRC incidence and CRC-related mortality subsequently, by interruption of the adenoma-carcinoma pathway [5–7]. Screening programmes can also reduce CRC-related mortality (but have no effect on incidence) by detecting asymptomatic cancers, which tend to be earlier-stage and thus associated with improved prognosis and survival. The impact of CRC screening is maximised in population-based programmes, which is more efficient and less costly than opportunistic screening [8].

Potential CRC screening tests can be generally divided into two categories: direct and indirect [9]. Stool-based tests (guaiac Faecal Occult Blood Test, gFOBT; Faecal Immunochemical Test, FIT; Faecal DNA tests) indirectly diagnose cancers and large adenomas by detecting their by-products (blood, DNA) in the stool. Such tests are non-invasive, and in the case of FBOT/FIT simple to perform and relatively cheap. However, because cancers may not bleed or only bleed intermittently, they need to be repeated frequently (e.g. every 2 years). Also, approximately 50 % may be false positives, leading to unnecessary referrals for subsequent colonoscopy [10, 11]. Perhaps most importantly, indirect tests favour cancer detection rather than adenomas, and so provide less opportunity to impact on cancer incidence.

Direct tests such as flexible sigmoidoscopy, colonoscopy, CTC directly visualise the target lesion, be it cancers or adenomas. Such tests can impact on both cancer mortality and incidence, and so need repeating only once per 5–10 years [12, 13]. Compared with indirect tests, direct tests are more invasive (making them more burdensome) and costlier.

Newer potential tests include capsule endoscopy, serum-based markers including serum proteomics, nuclear matrix proteins and serum DNA testing. None of these have been sufficiently tested in representative populations.

## Effect of screening on CRC-specific mortality

Both gFOBT and flexible sigmoidoscopy screening have been shown to decrease CRC-related mortality. Several randomised-controlled trials have shown that biennial gFOBT screening leads to a subsequent mortality reduction of 11–21 %, after a median follow-up of 10–13 years (five to six rounds); overall CRC mortality reduction is approximately 14 % after 10 years of screening [6, 14–17]. One large randomised-controlled trial showed that a single flexible sigmoidoscopy performed between 55 and 64 years of age led to an overall CRC mortality reduction of 31 % amongst invitees, rising to 42 % in those who attended [7]. These data prove that removal of benign adenomas reduces the incidence of subsequent colorectal cancer. It is therefore plausible that other screening techniques that reliably identify significant adenomas, namely colonoscopy and CTC, will also impact on CRC-related mortality. The magnitude of benefit can only be determined precisely via large randomised-controlled trials with long-term follow-up. The NordICC-trial (Nordic Initiative on Colorectal Cancer) is such a trial that evaluates the effect of colonoscopy screening on CRC-related mortality with its end-point at 10 years [18]. At the time of writing, we are aware of no current or planned studies that aim to evaluate the effect of CTC on CRC-related mortality.

## Accuracy

The diagnostic performance of various screening tests is summarised in Table 1. The gFOBT and FIT tests have a per-patient sensitivity of 11-20 % and 27–48 % respectively for advanced neoplasia and of 13–38 % and 56–88 % for CRC [19–23]. Sigmoidoscopy has a sensitivity of approximately 83 % for advanced neoplasia and 58–75 % for CRC [21, 24]. Colonoscopy has a sensitivity of 88 % for all advanced neoplasia, of 98 % for advanced neoplasia ≥10 mm, and of 95–97 % for CRC [21, 25, 26].

**Table 1:** Accuracy studies CTC screening, per patient sensitivity

Screening studies	Number of participants	All polyps		Adenomas	
		6-9 mm	≥10 mm	6-9 mm	≥10 mm
Graser et al. [21]	307	86.2	92. 6	90.5	92.0
Johnson et al. [28]	2,249	60.5	80.4	67.4	83.3
Kim et al. [29]	229	60.5	86.7	66.7	90.0
Macari et al. [30]	68	n.a.	n.a.	n.a.	100
Pickhardt et al. [26, 31]	1,233	n.a.	n.a.	86.7	93.8

Data are derived from the meta-analysis on CTC screening by De Haan et al. [27], and do not always correspond directly to the data that were provided in the original articles because: (1) high-risk participants were excluded from the analyses [28, 29], (2) a different matching algorithm was used in one study [28], or because additional data (i.e. on accuracy for all polyps) were collected [21, 28, 29].

*n.a.* not available

CTC might be a viable alternative since it has an estimated per-patient sensitivity of 88 % for advanced neoplasia ≥10 mm in screening populations [27]. These estimates are based on the aggregated results of five studies (*n* = 4,086 average risk participants, Table 1) that each evaluated the sensitivity of CTC relative to colonoscopy in a screening population [21, 26, 28–31]. Three of these studies reported per-patient sensitivity for advanced neoplasia ≥6 mm, ranging from 84 % to 93 % [21, 28, 29]. None of the six CRCs were missed by CTC. A recently published meta-analysis (including both average- and high-risk subjects) found that CTC has a sensitivity of 96 % for CRC, which is comparable to colonoscopy [32].

## Attendance and diagnostic yield

The efficacy of a screening programme is not only determined by diagnostic test accuracy but also by the proportion of invitees who ultimately attend. For example, the diagnostic yield of CRC screening can be defined as the number of invitees ultimately found to have advanced neoplasia, per 100 invitees.

Several trials have determined the attendance and subsequent diagnostic yield of gFOBT and FIT screening [33–35]. In most studies, invitees were asked to undergo more than one test, a procedure that may underestimate attendance compared with the offer of a single test. In one of these studies, 23 % of kits that were distributed by the community drug stores (containing three different stool tests) were completed and returned [33]. An unrandomised invitational-based Australian study, in which invitees were offered the choice of gFOBT, FIT, or both, found that 36 % of invitees participated overall, but relatively more participants participated in FIT screening than gFOBT (OR, 1.9; 95 % CI, 1.6–2.2) [35]. The gFOBT and FIT tests had different diagnostic yields for advanced neoplasia of approximately 1.4 vs 3.3 per 100 participants respectively (0.4 vs 1.3 per 100 invitees), but this difference disappeared after adjustment for differences in baseline characteristics.

In The Netherlands, several population-based screening trials have been performed over the last decade, comparing attendance and subsequent yield of a first screening round for gFOBT, FIT and/or flexible sigmoidoscopy [36, 37]. The gFOBT and FIT tests had participation rates of 47 % and 59–60 %, figures that are relatively high compared with those from other studies [33–35, 38]. Contrasting with data from previous studies, FIT screening resulted in higher attendance rates than gFOBT. Thirty percent of invitees for flexible sigmoidoscopy participated [36]. However, despite higher participation rates for gFOBT and FIT over flexible sigmoidoscopy, first round diagnostic yield was only 0.6 and 1.4–1.5 per 100 invitees, compared with 2.2 for sigmoidoscopy [36, 37].

Previous studies of flexible sigmoidoscopy screening, performed in Italy and the UK, randomised invitees after they had indicated that they were interested in participation or after preselection of average risk subjects by the general practitioner (in order to increase power) preventing precise evaluation of attendance rates [7, 39–42]. In the UK study, 38 % of citizens that were contacted participated, while initially 55 % of subjects responded positively to a mailed questionnaire that they would like to attend if invited.

As far as we are aware, two randomised controlled trials have been performed previously, in which the attendance and diagnostic yield of population-based CTC screening was compared with colonoscopy [43, 44]. An Australian trial reported participation rates of 18 % and 16 % for CTC and colonoscopy respectively, and a subsequent diagnostic yield (defined as number of participants with advanced neoplasia) of 9.0 and 8.4 per 100 participants [43]. A Dutch trial reported participation rates for CTC and colonoscopy of 34 % and 22 % respectively, and a subsequent diagnostic yield of 6.1 and 8.7 per 100 participants [44]. Ultimately, enhanced participation for CTC was countered by the greater sensitivity of colonoscopy, with the result that the two tests had similar diagnostic yields for advanced neoplasia of 2.2 and 1.9 per 100 invitees respectively. The Dutch trial differed in that participants undergoing CTC were only referred for subsequent colonoscopy if lesions ≥10 mm were detected (thus increasing positive predictive value); participants with lesions of 6–9 mm were offered surveillance with CTC [44]. Surveillance data are not yet available.

Table 2 compares attendance and yield for differing screening techniques when used in comparable settings and populations. Whether the differences in compliance and yield observed in the Dutch trials can be sustained and extrapolated into future screening rounds is currently unknown. Two studies reporting the attendance and yield of subsequent gFOBT screening rounds found that CRC detection rate decreased [45, 46]. The first study found that attendance decreased significantly from 59 % in the first round to 52 % in the second [45]. Although detection rates for advanced neoplasia were similar, cancer detection rates decreased significantly in the second round (from 1.35 to 0.94 per 1,000 screened) [45]. The second study found that the attendance rate was similar in the first, second and third rounds of gFOBT screening (55 %, 53 % and 55 %, respectively), but cancer detection decreased from 2.1 per 1,000 participants to 0.7 (first vs third round) [46].

**Table 2:** CRC screening techniques: overview of sensitivity, (Dutch) attendance and diagnostic yield of a first round of population-based screening, and CRC-related mortality reduction

	gFOBT	FIT	Sigmoidoscopy	Colonoscopy	CTC
Sensitivity					
- advanced neoplasia	11-20 % [19–23]	27-48 % [19–23]	83 % [21, 24]	88 % [21, 25, 26]	84-93 % [21, 26–32]^b^
- CRC	13-38 %	56-88 %	58-75 %	95-97 %	96-100 %
Attendance (%)^a^	47 % [36, 37]	59-60 % [36, 37]	30 % [36]	22 % [44]	34 % [44]
Yield advanced neoplasia					
- *per 100 exams*	1.2 [36, 37]	2.4-2.5 [36, 37]	7.3 [36]	8.7 [44]	6.1 [44]
- *per 100 invitees*	0.6	1.4-1.5	2.2	1.9	2.1
Mortality reduction	14 % [6]	Unknown	32 % [7]	Unknown	Unknown

^a^Attendance defined as number of invitees that completed the screening procedure

^b^Advanced neoplasia ≥6 mm

## CRC screening guidelines

Recently, the European Union (EU) recommended CRC screening for men and women aged 50–74 years [8]. Outside the EU, several organisations also recommend CRC screening, including the American Society for Gastrointestinal Endoscopy [47], the American Cancer Society, the US Multi-Society Task Force on Colorectal Cancer, the American College of Radiology [48, 49], the American College of Gastroenterology [50], and the Asia Pacific Working Group on Colorectal Cancer [51]. The societal benefits of CRC screening are clearly widely accepted. However, at this point in time there is no consensus regarding the preferred screening investigation or combination of modalities: the EU only specifies FOBT (gFOBT or FIT) as recommended screening tests since, at the time the guideline was drafted, gFOBT was the only test for which a significant decrease in disease-specific mortality had been demonstrated. Most of the US guidelines recommend colonoscopy as the first choice strategy based on superior sensitivity and specificity for adenomas and cancer (and ignore the fact that a reduction in disease-specific mortality has not been demonstrated directly); flexible sigmoidoscopy and/or CTC are alternative options for those patients who refuse colonoscopy or whose colonoscopy is incomplete. In Asia, either gFOBT, FIT, flexible sigmoidoscopy or colonoscopy screening are recommended. According to EU and Asian guidelines CTC is not recommended as a screening investigation due to insufficient evidence of efficacy.

At the time of writing, national CRC screening programmes for CRC have been implemented in several European countries. For example, in Finland, France and the UK gFOBT is used as the primary investigation. In Italy both FIT and flexible sigmoidoscopy are used as primary modalities, while in Poland only colonoscopy is used [8, 52]. The UK is currently implementing flexible sigmoidoscopy alongside the gFOBT programme, with a once-once examination offered at the ages of 55 years [53]. CTC has supplanted barium enema as the preferred whole-colon examination in gFOBT-positive patients who cannot undergo colonoscopy or whose colonoscopy is incomplete, as a consequence of results from a randomised-controlled trial of 5,427 symptomatic patients that found CTC significantly more sensitive than barium enema, with a lesser false-negative rate.

## Is there a role for CTC in population-based CRC screening?

### Advantages of CTC screening

As for colonoscopy, one of the major advantages postulated for CTC is that it enables visualisation of the entire colorectum. Secondly, CTC has the advantage that it detects advanced neoplasia in an early phase. CTC screening results in a higher diagnostic yield per 100 invitees than primary gFOBT and FIT screening, and in a similar yield as sigmoidoscopy and colonoscopy screening [41, 42, 44]. Because of enhanced detection characteristics for adenomas and cancers the inter-test interval for CTC is much longer than for stool-based tests; e.g once per 5–10 years versus once per 2 years [13]. Kim et al. [13] recently showed that in an audit of 1,011 screening participants with a negative baseline CTC, a single carcinoma occurred during an average follow-up period of 4.73 ± 1.15 years. Follow-up was primarily performed by reviewing the electronic medical records of all participants.

Several comparative non-randomised studies have shown that CTC is less burdensome than colonoscopy [54, 55]. A single randomised study of 547 patients performed by Von Wagner et al. (manuscript recently accepted by Radiology) found that CTC was more acceptable than colonoscopy and associated with less physical side-effects, but this was performed in a symptomatic setting (data derived by personal communication). Although most CTC studies have utilised full cathartic bowel cleansing, non-cathartic preparations are increasingly popular and are expected to enhance compliance. Iodine is already used to tag residual stool with the aim of facilitating radiological interpretation, and this approach can be extrapolated into a non-cathartic preparation, decreasing the volume ingested orally. A disadvantage of most iodinated contrast agents is that they induce diarrhoea because most agents are hyperosmotic. This non-cathartic bowel preparation has recently been shown in a randomised-controlled design to be less burdensome than the alternative of 2 l of laxatives and 2 l of clear fluids required for colonoscopy [56]. However, the same investigators also found that increased diarrhoea was perceived as more burdensome by CTC participants [56]. Invitees were also asked—before they underwent the allocated screening procedure—whether they anticipated the procedure would be burdensome: 36 % of colonoscopy invitees anticipated a “rather” or “extremely” burdensome experience compared with only 9 % of CTC invitees. Ultimately, 21 % of CTC participants indicated that the experience was “worse” than expected, compared with 12 % of colonoscopy participants. These findings suggest that CTC invitees underestimate the burden of CTC (e.g. diarrhoea and abdominal pain) relative to colonoscopy. The differences in perceived burden of the entire screening procedure were small (mean score on five-point Likert scale 1.8 in colonoscopy vs 2.0 in CTCl, *P* < 0.001), and did not result in a significantly different proportion of participants that indicated they would be willing to participate in a future screening round. Other bowel preparation schemes with less iodine may result in a less burdensome procedure.

The relatively low risk of serious adverse events associated with CTC is frequently cited as an advantage compared with colonoscopy screening, which has a complication risk of 0.1-0.3 % [57–59]. However, while this holds true for the examinations in isolation, the correct approach is to consider the diagnostic pathway as a whole. Colonoscopic complications precipitated by a positive CTC result must be included and accounted for as part of the diagnostic trajectory for screening CTC. In the Dutch trial, the prevalence of post-polypectomy bleeding was non-significantly different for CTC (0.3 % of participants) and colonoscopy (0.2 % of participants) [44].

Whether the visualisation of extracolonic structures and consequent detection of potential pathology is an advantage or disadvantage is frequently debated and remains unclear. It is clear, however, that potential screenees regard this aspect of CTC as attractive, intriguing and a distinct advantage over all other competing tests [60]. In a screening population, the prevalence of (potentially) important findings that precipitate additional diagnostic follow-up testing ranges from 4.5 to 11 % [30, 43, 61, 62]. One audit of 10,286 participants undergoing CTC screening found extracolonic (non-colorectal) cancer in 36 (0.35 %) participants; renal cell carcinoma, lung adenocarcinoma and non-Hodgkin lymphoma were most common [63]. However, the majority of potentially important findings ultimately emerge as clinically unimportant after follow-up testing, and therefore have the potential to cause anxiety, morbidity (and even mortality) for no clinical benefit. Furthermore, the incremental costs of diagnostic follow-up tests, including surgery and additional clinic visits, etc., may be substantial, averaging from €20 to €25 per participant overall [61, 62]. It is possible that early detection of important extracolonic findings might ultimately lead to lower costs and decreased mortality in the long run, but these data are currently unavailable and would require randomised trials with tens of thousands of participants. Currently, the only data available arise from modelling the costs and consequences of extracolonic detections using currently available research. For example, one approach is to screen only for intracolonic lesions, aortic aneurysms and extracolonic cancers [64, 65]. At the time of writing, it is uncertain whether this is feasible; in some countries, it is hard to imagine that radiologists will be allowed legally to “close their eyes” to certain categories of findings potentially revealed by CTC.

### Disadvantages of CTC screening

When CTC is used for screening—as for positive stool tests and flexible sigmoidoscopy—there is a need for subsequent testing in positive patients who have a potential lesion large enough to trigger subsequent colonoscopy. In an non-randomised USA study that evaluated the diagnostic yield per 100 participants for colonoscopy and CTC, 7.9 % of participants having CTC were referred for subsequent colonoscopy [57]. Participants with lesions of 6–9 mm were offered the choice of colonoscopy or surveillance CTC. Within the Dutch screening trial, 8.6 % of CTC participants were referred for colonoscopy as a consequence of lesions ≥10 mm. However, if a referral threshold of ≥6 mm were used, 16.7 % of participants having CTC would have been referred for subsequent colonoscopy.

Exposure to ionizing radiation may provoke radiation-induced cancers, but this potential disadvantage needs to be balanced against potential gains. Gonzales et al. [66] estimated that a 5-yearly CTC screening program would prevent the development of 24 CRCs for every radiation-induced cancer, based on an estimated mean effective dose of 8 mSv for women and 7 mSv for men. In fact, the dose conveyed by screening CTC averages 4 mSv [67].

Most previous cost-effectiveness models of population-based CTC screening programmes estimate that CTC is less cost-effective than alternative methods [64, 68–73]. According to some of these models, CTC screening could be more cost-effective than colonoscopy if the unit cost per CTC falls to less than 60-72 % of the unit costs for colonoscopy [64, 68], if attendance for CTC is ≥25 % higher than colonoscopy [69] or a combination of both that offers a net-benefit [70]. However, the precise unit cost of CTC when employed within population-based CRC screening programmes is largely unknown, and will vary depending on the healthcare system in question. Existing estimates (calculated in previous cost-effectiveness analyses) are based on unit costs ranging from €346 to €594 for abdominal and/or pelvic CT or colonoscopy, or on unspecified assumptions related to costs [64, 65, 68–73]. Increased efficiencies that are likely to accompany the deployment of CTC in a screening programme would probably diminish the unit cost for CTC when used in this setting, compared with unit costs in symptomatic patients. Ultimately, the key-metric required by health policy makers will be the cost-per-cancer detected and the cost-per-significant-adenoma detected per 100 subjects invited for screening, which allows direct cost-comparisons between competing screening modalities. The costs, diagnoses and consequences of screening should be collected prospectively as part of a randomised controlled trial (or established screening programme) so that the cost-effectiveness model is populated with reliable data rather than test characteristics and costs estimated from the literature, which may be inaccurate or derived from healthcare settings that are not generalizable to the setting in question.

### Logistics

Before CTC is implemented as the primary screening investigation, sufficient CT system capacity should be available. Assuming that there is limited free time available on hospital CT systems currently, this would likely require large-scale investment to increase available screening capacity. One potential approach might utilise mobile CTC units similar to those that are used currently for breast cancer screening in several countries, including the UK and The Netherlands; such dedicated units are likely efficient and may increase attendance due to the convenience of proximity. It is clear that sufficient, adequately trained CTC readers are needed; on average, inexperienced readers need training with at least 175 individual cases before they reach an acceptable sensitivity and specificity for lesions of 6 mm and larger [74]. One alternative possibility is to train radiographic technicians to interpret CTC, since they are less expensive than radiologists [75]. Such an approach would also lower the unit cost of CTC. Previously, several studies in clinical cohorts had indicated that technologists may be adequate CTC readers, as they reached a diagnostic accuracy that was comparable to the radiologist [76–78]. A recent study performed within a population-based CTC screening study showed that a reading strategy of two technologists with or without consensus would result in a comparable diagnostic yield for advanced neoplasia compared with a reading strategy of one radiologist [79].

Another alternative could be to use computer-aided detection (CAD) as second reader, subsequent to the radiologist’s interpretation. This strategy has shown to result in significantly higher sensitivity for lesions of ≥6 mm and although specificity inevitable decreases, it does not do so significantly [75, 80]. Additionally, CAD might be used as the primary CTC reader (i.e. in advance of any radiologist interpretation), followed by a radiologist evaluation restricted to the CAD marks. An Italian study of FOBT positives recently showed that such a reading detected 11 of 13 colorectal carcinomas. If the evaluation of CAD findings was followed by evaluation of the 2D images, this resulted in a sensitivity of 89 % for advanced adenomas, which was comparable to double primary 2D read followed by secondary CAD read [81].

## CTC as a triage test following positive FOBT?

As positive stool tests have a relatively limited positive predictive value for CRC, it has been suggested that CTC could be used as an intermediate test, triaging FOBT positives for colonoscopy. CTC has a per-patient sensitivity of 93 % for adenomas ≥6 mm and of 95 % for advanced neoplasia ≥10 mm and CRC, in FOBT positives (using FIT or gFOBT) [82]. A previous study performed in 302 FOBT-positive individuals found that CTC would prevent a subsequent colonoscopy in only 28 % of cases, while lesions ≥10 mm would have been missed in 2 % [83]. Given the relatively high prevalence of abnormality in patients who are FOBT-positive, it is unlikely that CTC is a clinically effective or cost-effective triage method in first-round FOBT screening. CTC may have a role in subsequent rounds when the prevalence of abnormality may be expected to decrease, or where newer stool tests are employed that have greater sensitivity but proportionally less specificity when compared with gFOBT.

## Conclusion

Screening for colorectal cancer using CTC as a primary investigation is feasible; the detection characteristics of CTC in this context are increasingly well-established and compliance seems to be increased. These factors lead to a similar yield of advanced neoplasia for CTC compared with colonoscopy and flexible sigmoidoscopy, and higher yields than gFOBT and FIT. Therefore, the viability of CTC as a primary screening investigation is likely to turn on other factors, such as cost-effectiveness and whether the ability to detect extracolonic disease (impossible with alternative investigations) is ultimately beneficial or not. Whether there is a need to demonstrate a direct reduction in disease-specific mortality is debatable given that detection characteristics of CTC for cancers and adenomas are well-defined. However, the CTC community should be aware that the lack of large-scale implementation pilots, including an evaluation of effect on disease-specific mortality, remains an obstacle for health-policy makers shaping population-based CTC screening in Europe in the future.

## Abbreviations and acronyms

CRC: Colorectal cancer
CT: Computed tomography
EU: European Union

## References

[1] Ferlay J, Autier P, Boniol M, Heanue M, Colombet M, Boyle P (2007). Estimates of the cancer incidence and mortality in Europe in 2006. Ann Oncol.

[2] http://globocan.iarc.fr/ (accessed January 9, 2012)

[3] Bond JH (2000). Clinical evidence for the adenoma-carcinoma sequence, and the management of patients with colorectal adenomas. Semin Gastrointest Dis.

[4] Morson BC (1974). Evolution of cancer of the colon and rectum. Cancer.

[5] Winawer SJ, Zauber AG, Ho MN (1993). Prevention of colorectal cancer by colonoscopic polypectomy. The National Polyp Study Workgroup. N Engl J Med.

[6] Heresbach D, Manfredi S, D'Halluin PN, Bretagne JF, Branger B (2006). Review in depth and meta-analysis of controlled trials on colorectal cancer screening by faecal occult blood test. Eur J Gastroenterol Hepatol.

[7] Atkin WS, Edwards R, Kralj-Hans I (2010). Once-only flexible sigmoidoscopy screening in prevention of colorectal cancer: a multicentre randomised controlled trial. Lancet.

[8] http://screening.iarc.fr/doc/ND3210390ENC.pdf (accessed January 9, 2012)

[9] de Wijkerslooth TR, Bossuyt PM, Dekker E (2011). Strategies in screening for colon carcinoma. Neth J Med.

[10] Allison JE, Sakoda LC, Levin TR (2007). Screening for colorectal neoplasms with new fecal occult blood tests: update on performance characteristics. J Natl Cancer Inst.

[11] Hewitson P, Glasziou P, Watson E (2008). Cochrane systematic review of colorectal cancer screening using the fecal occult blood test (hemoccult): an update. Am J Gastroenterol.

[12] Brenner H, Chang-Claude J, Seiler CM, Sturmer T, Hoffmeister M (2006). Does a negative screening colonoscopy ever need to be repeated?. Gut.

[13] Kim DH, Pooler BD, Weiss JM, Pickhardt PJ (2011) Five year colorectal cancer outcomes in a large negative CT colonography screening cohort. Eur Radiol. doi:10.1007/s00330-011-2365-2 10.1007/s00330-011-2365-2 PMC3358445 22210409

[14] Mandel J, Church J, Ederer F, Bond J (1999). Colorectal cancer mortality: effectiveness of biennial screening for fecal occult blood. J Natl Cancer Inst.

[15] Scholefield J, Moss S, Sufi F, Mangham C, Hardcastle J (2002). Effect of faecal occult blood screening on mortality from colorectal cancer: results from a randomised controlled trial. Gut.

[16] Faivre J, Dancourt V, Lejeune C (2004). Reduction in colorectal cancer mortality by fecal occult blood screening in a French controlled study. Gastroenterology.

[17] Kronborg O, Jorgensen O, Fenger C, Rasmussen M (2004). Randomized study of biennial screening with a faecal occult blood test: results after nine screening rounds. Scand J Gastroenterol.

[18] http://www.kreftregisteret.no/PageFiles/2151/NordICC%20protocol%20MB_270510.pdf Accessed January 9, 2012

[19] Imperiale TF, Ransohoff DF, Itzkowitz SH, Turnbull BA, Ross ME (2004). Fecal DNA versus fecal occult blood for colorectal-cancer screening in an average-risk population. N Engl J Med.

[20] Cheng TI, Wong JM, Hong CF (2002). Colorectal cancer screening in asymptomatic adults: comparison of colonoscopy, sigmoidoscopy and fecal occult blood tests. J Formos Med Assoc.

[21] Graser A, Stieber P, Nagel D (2009). Comparison of CT colonography, colonoscopy, sigmoidoscopy and faecal occult blood tests for the detection of advanced adenoma in an average risk population. Gut.

[22] Morikawa T, Kato J, Yamaji Y, Wada R, Mitsushima T, Shiratori Y (2005). A comparison of the immunochemical fecal occult blood test and total colonoscopy in the asymptomatic population. Gastroenterology.

[23] Nakama H, Yamamoto M, Kamijo N (1999). Colonoscopic evaluation of immunochemical fecal occult blood test for detection of colorectal neoplasia. Hepatogastroenterology.

[24] http://www.gezondheidsraad.nl/nl/adviezen/bevolkingsonderzoek-naar-darmkanker (accessed January 5, 2012)

[25] van Rijn JC, Reitsma JB, Stoker J, Bossuyt PM, van Deventer SJ, Dekker E (2006). Polyp miss rate determined by tandem colonoscopy: a systematic review. Am J Gastroenterol.

[26] Pickhardt PJ, Choi JR, Hwang I (2003). Computed tomographic virtual colonoscopy to screen for colorectal neoplasia in asymptomatic adults. N Engl J Med.

[27] de Haan MC, van Gelder RE, Graser A, Bipat S, Stoker J (2011). Diagnostic value of CT-colonography as compared to colonoscopy in an asymptomatic screening population: a meta-analysis. Eur Radiol.

[28] Johnson CD, Chen MH, Toledano AY (2008). Accuracy of CT colonography for detection of large adenomas and cancers. N Engl J Med.

[29] Kim YS, Kim N, Kim SH (2008). The efficacy of intravenous contrast-enhanced 16-raw multidetector CT colonography for detecting patients with colorectal polyps in an asymptomatic population in Korea. J Clin Gastroenterol.

[30] Macari M, Bini EJ, Jacobs SL (2004). Colorectal polyps and cancers in asymptomatic average-risk patients: evaluation with CT colonography. Radiology.

[31] Pickhardt PJ, Choi JR, Hwang I, Schindler WR (2004). Nonadenomatous polyps at CT colonography: prevalence, size distribution, and detection rates. Radiology.

[32] Pickhardt PJ, Hassan C, Halligan S, Marmo R (2011). Colorectal cancer: CT colonography and colonoscopy for detection–systematic review and meta-analysis. Radiology.

[33] Petrelli N, Michalek AM, Freedman A (1994). Immunochemical versus guaiac occult blood stool tests: results of a community based screening program. Surg Oncol.

[34] Robinson MH, Marks CG, Farrands PA (1994). Population screening for colorectal cancer: comparison between guaiac and immunological faecal occult blood tests. Br J Surg.

[35] Hughes K, Leggett B, Del Mar C (2005). Guaiac versus immunochemical tests: faecal occult blood test screening for colorectal cancer in a rural community. Aust N Z J Public Health.

[36] Segnan N, Senore C, Andreoni B (2005). Randomized trial of different screening strategies for colorectal cancer: patient response and detection rates. J Natl Cancer Inst.

[37] Segnan N, Senore C, Andreoni B (2007). Comparing attendance and detection rate of colonoscopy with sigmoidoscopy and FIT for colorectal cancer screening. Gastroenterology.

[38] Hardcastle J, Chamberlain J, Robinson M (1996). Randomised controlled trial of faecal-blood screening for colorectal cancer. Lancet.

[39] Segnan N, Senore C, Andreoni B (2002). Baseline findings of the Italian multicenter randomized controlled trial of “once-only sigmoidoscopy”—SCORE. J Natl Cancer Inst.

[40] UK Flexible Sigmoidoscopy Screening Trial Investigators (2002). Single flexible sigmoidoscopy screening to prevent colorectal cancer: baseline findings of a UK multicentre randomised trial. Lancet.

[41] Hol L, van Leerdam ME, van Ballegooijen M (2010). Screening for colorectal cancer: randomised trial comparing guaiac-based and immunochemical faecal occult blood testing and flexible sigmoidoscopy. Gut.

[42] van Rossum LG, van Rijn AF, Laheij RJ (2008). Random comparison of guaiac and immunochemical fecal occult blood tests for colorectal cancer in a screening population. Gastroenterology.

[43] Scott RG, Edwards JT, Fritschi L (2004). Community-based screening by colonoscopy or computed tomographic colonography in asymptomatic average-risk subjects. Am J Gastroenterol.

[44] Stoop EM, de Haan MC, de Wijkerslooth TR (2011). Participation and yield of colonoscopy versus non-cathartic CT colonography in population-based screening for colorectal cancer: a randomised controlled trial. Lancet Oncol.

[45] Weller D, Coleman D, Robertson R (2007). The UK colorectal cancer screening pilot: results of the second round of screening in England. Br J Cancer.

[46] Steele RJ, McClements PL, Libby G (2009). Results from the first three rounds of the Scottish demonstration pilot of FOBT screening for colorectal cancer. Gut.

[47] Davila RE, Rajan E, Baron TH (2006). Standards of Practice Committee. American Society for Gastrointestinal Endoscopy. ASGE guideline: colorectal cancer screening and surveillance. Gastrointest Endosc.

[48] Levin B, Lieberman DA, McFarland B (2008). Screening and surveillance for the early detection of colorectal cancer and adenomatous polyps, 2008: a joint guideline from the American Cancer Society, the US Multi-Society Task Force on Colorectal Cancer, and the American College of Radiology. Gastroenterology.

[49] McFarland EG, Levin B, Lieberman DA (2008). American Cancer Society; U.S. Multisociety Task Force on Colorectal Cancer; American College of Radiology. Revised colorectal screening guidelines: joint effort of the American Cancer Society, U.S. Multisociety Task Force on Colorectal Cancer, and American College of Radiology. Radiology.

[50] Rex DK, Johnson DA, Anderson JC (2009). American College of Gastroenterology. American College of Gastroenterology guidelines for colorectal cancer screening. Am J Gastroenterol.

[51] Sung JJ, Lau JY, Young GP (2008). Asia Pacific Working Group on Colorectal Cancer. Asia Pacific consensus recommendations for colorectal cancer screening. Gut.

[52] http://ec.europa.eu/health/archive/ph_determinants/genetics/documents/cancer_screening.pdf

[53] http://www.cancerscreening.nhs.uk/bowel/flexible-sigmoidoscopy-screening.html

[54] Ristvedt SL, McFarland EG, Weinstock LB (2003). Patient preferences for CT colonography, conventional colonoscopy, and bowel preparation. Am J Gastroenterol.

[55] Jensch S, Bipat S, Peringa J (2010). CT colonography with limited bowel preparation: prospective assessment of patient experience and preference in comparison to optical colonoscopy with cathartic bowel preparation. Eur Radiol.

[56] de Wijkerslooth TR, de Haan MC, Stoop EM et al (2011) Burden of colonoscopy compared to non-cathartic CT-colonography in a colorectal cancer screening programme: randomised controlled trial. Gut. doi:10.1136/gutjnl-2011-301308 10.1136/gutjnl-2011-301308 22198714

[57] Kim DH, Pickhardt PJ, Taylor AJ (2007). CT colonography versus colonoscopy for the detection of advanced neoplasia. N Engl J Med.

[58] Panteris V, Haringsma J, Kuipers EJ (2009). Colonoscopy perforation rate, mechanisms and outcome: from diagnostic to therapeutic colonoscopy. Endoscopy.

[59] Nelson DB, McQuaid KR, Bond JH, Lieberman DA, Weiss DG, Johnston TK (2002). Procedural success and complications of large-scale screening colonoscopy. Gastrointest Endosc.

[60] Von Wagner C, Smith S, Magee MS, Halligan S, Wardle J (2011). Preferences for invasive and non-invasive colorectal cancer screening tests: using a structured group discussion to elicit in-depth commentary and individual preferences. Ann Behav Med Springer.

[61] Yee J, Kumar NN, Godara S (2005). Extracolonic abnormalities discovered incidentally at CT colonography in a male population. Radiology.

[62] Gluecker TM, Johnson CD, Wilson LA (2003). Extracolonic findings at CT colonography: evaluation of prevalence and cost in a screening population. Gastroenterology.

[63] Pickhardt PJ, Kim DH, Meiners RJ (2010). Colorectal and extracolonic cancers detected at screening CT colonography in 10,286 asymptomatic adults. Radiology.

[64] Hassan C, Pickhardt PJ, Laghi A (2008). Computed tomographic colonography to screen for colorectal cancer, extracolonic cancer, and aortic aneurysm: model simulation with cost-effectiveness analysis. Arch Intern Med.

[65] Pickhardt PJ, Hassan C, Laghi A (2009). CT colonography to screen for colorectal cancer and aortic aneurysm in the Medicare population: cost-effectiveness analysis. AJR Am J Roentgenol.

[66] de González AB, Kim KP, Knudsen AB (2011). Radiation-related cancer risks from CT colonography screening: a risk-benefit analysis. AJR Am J Roentgenol.

[67] Liedenbaum MH, Venema HW, Stoker J (2008). Radiation dose in CT colonography—trends in time and differences between daily practice and screening protocols. Eur Radiol.

[68] Ladabaum U, Song K, Fendrick AM (2004). Colorectal neoplasia screening with virtual colonoscopy: when, at what cost, and with what national impact?. Clin Gastroenterol Hepatol.

[69] Knudsen AB, Lansdorp-Vogelaar I, Rutter CM (2010). Cost-effectiveness of computed tomographic colonography screening for colorectal cancer in the medicare population. J Natl Cancer Inst.

[70] Lansdorp-Vogelaar I, van Ballegooijen M, Zauber AG, Boer R, Wilschut J, Habbema JD (2009). At what costs will screening with CT colonography be competitive? A cost-effectiveness approach. Int J Cancer.

[71] Sonnenberg A, Delcò F, Bauerfeind P (1999). Is virtual colonoscopy a cost-effective option to screen for colorectal cancer?. Am J Gastroenterol.

[72] Vijan S, Hwang I, Inadomi J (2007). The cost-effectiveness of CT colonography in screening for colorectal neoplasia. Am J Gastroenterol.

[73] Vanness DJ, Knudsen AB, Lansdorp-Vogelaar I (2011). Comparative Economic Evaluation of Data from the ACRIN National CT Colonography Trial with Three Cancer Intervention and Surveillance Modeling Network Microsimulations. Radiology.

[74] Liedenbaum MH, Bipat S, Bossuyt PM (2011). Evaluation of a standardized CT colonography training program for novice readers. Radiology.

[75] Dachman AH, Obuchowski NA, Hoffmeister JW (2010). Effect of computer-aided detection for CT colonography in a multireader, multicase trial. Radiology.

[76] Jensch S, van Gelder RE, Florie J (2007). Performance of radiographers in the evaluation of CT colonographic images. AJR Am J Roentgenol.

[77] Burling D, Wylie P, Gupta A (2010). CT colonography: accuracy of initial interpretation by radiographers in routine clinical practice. Clin Radiol.

[78] Lauridsen C, Lefere P, Gerke O, Gryspeerdt S (2011) Effect of a tele-training programme on radiographers in the interpretation of CT colonography. Eur J Radiol. doi:10.1016/j.ejrad.2011.02.028 10.1016/j.ejrad.2011.02.028 21397422

[79] De Haan MC, Nio CY, Thomeer M, et al (2012) Comparing the diagnostic yield of technologists and radiologists in an invitational colorectal cancer screening program using CT-colonography. Radiology (in press) 10.1148/radiol.12112486 22771881

[80] Halligan S, Mallett S, Altman DG (2011). Incremental benefit of computer-aided detection when used as a second and concurrent reader of CT colonographic data: multiobserver study. Radiology.

[81] Iussich G, Correale L, Senore C et al (2011) CT colonography (CTC) per lesion sensitivity of a double-reading paradigm where CAD is the first reader. Presented at: Radiological Society of North America 2011 Scientific Assembly and Annual Meeting, Chicago IL. m.rsna.org/download.cfm?f=inform/

[82] Liedenbaum MH, de Vries AH, van Rijn AF (2010). CT colonography with limited bowel preparation for the detection of colorectal neoplasia in an FOBT positive screening population. Abdom Imaging.

[83] Liedenbaum MH, van Rijn AF, de Vries AH (2009). Using CT colonography as a triage technique after a positive faecal occult blood test in colorectal cancer screening. Gut.

